# Neurogenic Potential of the Vestibular Nuclei and Behavioural Recovery Time Course in the Adult Cat Are Governed by the Nature of the Vestibular Damage

**DOI:** 10.1371/journal.pone.0022262

**Published:** 2011-08-11

**Authors:** Sophie Dutheil, Michel Lacour, Brahim Tighilet

**Affiliations:** Département de Neurosciences, UMR 6149 “Neurosciences Intégratives et Adaptatives”, Université de Provence/CNRS - Pôle 3C (Comportement, Cerveau, Cognition), Centre de Saint Charles, Marseille, France; Consejo Superior de Investigaciones Cientificas, Spain

## Abstract

Functional and reactive neurogenesis and astrogenesis are observed in deafferented vestibular nuclei after unilateral vestibular nerve section in adult cats. The newborn cells survive up to one month and contribute actively to the successful recovery of posturo-locomotor functions. This study investigates whether the nature of vestibular deafferentation has an incidence on the neurogenic potential of the vestibular nuclei, and on the time course of behavioural recovery. Three animal models that mimic different vestibular pathologies were used: unilateral and permanent suppression of vestibular input by unilateral vestibular neurectomy (UVN), or by unilateral labyrinthectomy (UL, the mechanical destruction of peripheral vestibular receptors), or unilateral and reversible blockade of vestibular nerve input using tetrodotoxin (TTX). Neurogenesis and astrogenesis were revealed in the vestibular nuclei using bromodeoxyuridine (BrdU) as a newborn cell marker, while glial fibrillary acidic protein (GFAP) and glutamate decarboxylase 67 (GAD67) were used to identify astrocytes and GABAergic neurons, respectively. Spontaneous nystagmus and posturo-locomotor tests (static and dynamic balance performance) were carried out to quantify the behavioural recovery process. Results showed that the nature of vestibular loss determined the cellular plastic events occurring in the vestibular nuclei and affected the time course of behavioural recovery. Interestingly, the deafferented vestibular nuclei express neurogenic potential after acute and total vestibular loss only (UVN), while non-structural plastic processes are involved when the vestibular deafferentation is less drastic (UL, TTX). This is the first experimental evidence that the vestibular complex in the brainstem can become neurogenic under specific injury. These new data are of interest for understanding the factors favouring the expression of functional neurogenesis in adult mammals in a brain repair perspective, and are of clinical relevance in vestibular pathology.

## Introduction

In all mammals, unilateral damage of the peripheral vestibular system induces a post-lesional vestibular syndrome made of static and dynamic signs. The static signs include oculomotor deficits (spontaneous horizontal nystagmus) and postural deficits (head tilt, increased body support surface) that are compensated within a few days or weeks, while the dynamic signs (vestibulo-ocular reflex asymmetry, impaired equilibrium function) are compensated much less completely or over a longer time [1], [2], [3]. Immedialely after unilateral vestibular loss, ipsilateral vestibular nuclei (VN) are silenced whereas contralateral VN keep resting activity. Over time, behavioural deficits improve and compensate in a time window that approximately coincides with restoration of balanced electrical activity between the two sides [4]. This rebalanced activity within the VN is attributed to different cellular neuroplasticity mechanisms: molecular, neurochemical, and neurohormonal changes at pre- and post-synaptic levels, sensitivity changes of receptors, neuromediator release, sprouting of axon collaterals, and astroglial and microglial reactions [1], [3], [5], [6], [7], [8].

In addition to these plasticity mechanisms, we demonstrated that unilateral vestibular neurectomy (UVN) in the adult cat induced intense cell proliferation in the deafferented VN. Most of the reactive newborn cells survived up to one month, differentiated into astrocytic, microglial cells and GABAergic neurons [9], and contributed actively to the successful recovery of posturo-locomotor functions [10]. Such a structural plasticity mechanism after vestibular loss is of particular interest to understand the vestibular compensation process and the factors favouring the expression of reactive neurogenesis in the adult damaged brain.

Adult neurogenesis is an ongoing process restricted to two zones in the healthy mammal brain: the subventricular zone (SVZ) of the lateral ventricles and the subgranular zone (SGZ) of the dentate gyrus in the hippocampus. Pathological conditions such as Alzheimer disease [11], Huntington disease [12], Parkinson disease [13], seizures [14], strokes [15] modulate the neurogenesis rate in the SVZ and SGV. Furthermore, specific damages of the central nervous system can lead to reactive neurogenesis even in non-neurogenic zones – in the dorsal vagal complex [16], in the corticospinal pathway [17], in the hypothalamus [18], in the cortex [19], in the striatum [20]. The determinants of such a switch from non-neurogenic to a neurogenic area are still poorly known [21], [22].

In this study, we asked first whether the plasticity mechanisms involved in recovery after vestibular loss, including the astroneurogenesis reaction, depend on the nature of the vestibular damage. To answer this question, we designed three experimental groups of adult cats that underwent different vestibular lesions: one group was submitted to a unilateral and reversible blockade of vestibular input by intratympanic injection of tetrodrotoxin (TTX), a second group received a unilateral labyrinthectomy (UL) by destruction of the vestibular peripheral receptors in the inner ear, and a third group had a post-ganglion unilateral vestibular neurectomy (UVN). These three vestibular lesion models induce different type of VN deafferentation which should induce different neurobiological plasticity reactions: sudden and total structural and functional deafferentation after UVN (like in vestibular neuritis and Menière's disease surgery), more progressive and mainly functional deafferentation after UL (like in ototoxic drug administration, ageing), transient and reversible deafferentation after TTX (a model more or less close to benign paroxysmal positional vertigo, BPPV). We used bromodeoxyuridine (BrdU) as a newborn cell marker, glial fibrillary acidic protein (GFAP) as an astrocyte marker, and glutamate decarboxylase 67 (GAD67) as an indicator of GABAergic phenotype. Our working hypothesis was that the neurogenesis reaction should be expressed mainly after sudden and acute vestibular loss (UVN). Immunohistochemistry methods served to reveal both changes of plasticity markers expression from existing cells and the presence of newly generated astrocytes and GABAergic neurons. A second aim of the study was to determine whether these three models of vestibular deafferentation resulted in different time courses of behavioural recovery. For this purpose, we analysed the compensation of the spontaneous vestibular nystagmus and of the posturo-locomotor syndrome (support surface, locomotor equilibrium) as a function of time after vestibular damage. Finally, this experimental study opens widely to the question: do patients with vestibular pathologies of different aetiologies recover using different plasticity mechanisms?

## Results

### Cell proliferation analysis

**Figure 1:**
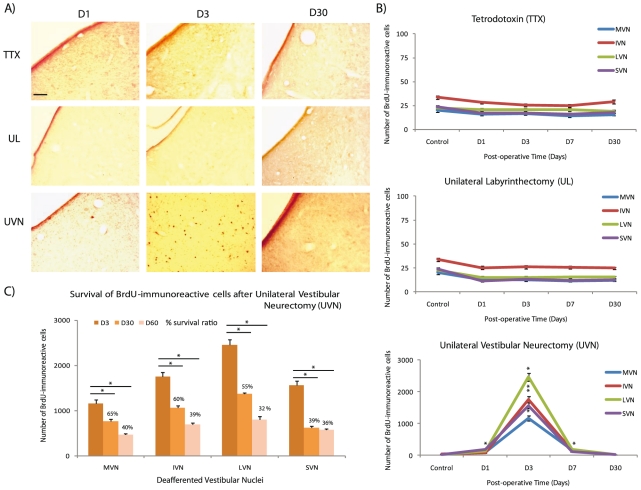
Reactive cell proliferation depends on the nature of the vestibular deafferentation. (**A**) Representative illustrations of 5-bromo-2′-deoxyuridine (BrdU) immunostainings in the deafferented MVN in control cats and in cats examined 1, 3, and 30 days after unilateral tetrodotoxin injection (TTX), unilateral labyrinthectomy (UL), and unilateral vestibular neurectomy (UVN). Scale bar: 100 *µ*m. (**B**) Quantitative evaluation. Curves illustrating the mean values (± SEM, vertical bars) in the deafferented VN in control cats and in cats subjected to TTX, UL, and UVN and observed at 1, 3, 7, and 30 days after the different vestibular deafferentations. * Indicates significant differences assessed by ANOVA followed by Scheffé test for all the VN (p<0.0001). (**C**) Survival of the newly generated cells 30 and 60 days after UVN. Histograms comparing the mean values (± SEM) of the number of BrdU-immunopositive cells in the deafferented VN 3, 30 and 60 day after UVN. BrdU was injected 3 days after UVN. *Significant differences assessed by ANOVA followed by Scheffé test (p<0.0001). This comparison served to establish a survival ratio specific to each VN. Only values recorded on the lesioned side are illustrated. BrdU: 5-bromo-2′deoxyuridine; D: day; IVN: inferior vestibular nucleus; LVN: lateral vestibular nucleus; MVN: medial vestibular nucleus; SVN: superior vestibular nucleus; TTX: tetrodoxin; UL: unilateral labyrinthectomy, UVN: unilateral vestibular neurectomy. *n = *4 animals per group.

As recently demonstrated [9], [10] control animals did not exhibit BrdU-positive (BrdU^+^) cells in the whole VN and around the subependimary layer of the fourth ventricle, whereas UVN induced strong and notable cell proliferation restricted to the deafferented VN. The quantification of the BrdU^+^ cells in the VN of UVN animals reproduced a pattern of expression similar to that in previous studies, with stronger cell proliferation in LVN and IVN than in MVN and SVN [9] (Figures 1A and 1B). Cell proliferation began 1 day after UVN, reached a peak at 3 days (+5671.14% in the MVN, +5605.88% in the IVN, +10536.36% in the LVN and +6454.62% in the SVN compared to controls), and then decreased to reach control values 30 days after UVN. Most of the newborn cells that peaked at D3 survived up to 2 months after UVN (Figure 1C) and integrated functional networks [10]. By contrast, in unilateral labyrinthectomy (UL) and TTX groups, very slight and non-significant numbers of BrdU^+^ cells were detected in the VN whatever the post-lesion delay (D1, D3, D7, D30 and D60) and the side considered (ipsi- or contra-lateral). Statistical analysis did not display significant difference between these three groups (TTX versus UL versus controls).

### GAD67 expression in the deafferented vestibular nuclei

**Figure 2:**
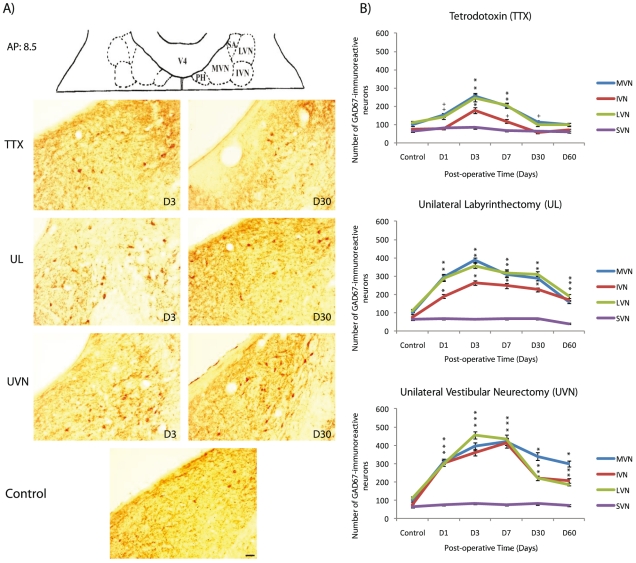
Glutamic acid decarboxylase (GAD67) immunoreactivity is differentially expressed according to the vestibular damage. (**A**) Schematic representation illustrating the localization of the antero-posterior (AP) axis adapted from Berman's strereotaxis Atlas (1968), where the photomicrographs of the medial vestibular nuclei were taken. PH: praepositus hypoglossi; SA: stria acustica; V4: fourth ventricle. GAD67-immunoreactive neurons are expressed in all the vestibular nuclei. Illustration of GAD67 immunoreactivity in the medial vestibular nucleus (MVN) in a representative control cat and in three experimental animals examined 3 and 30 days after TTX injection, unilateral labyrinthectomy (UL), or unilateral neurectomy (UVN). (**B**) Quantitative evaluation of the effects of the nature of vestibular deafferentation (TTX, UL, or UVN) on GAD67-immunoreactive neurons in the deafferented vestibular nuclei. Data are mean values (± SEM) of the number of GAD67-immunoreactive neurons in the deafferented vestibular nuclei of control cats and cats in the experimental groups. Only values recorded on the lesioned side are illustrated. Data from both sides of control cats were pooled for direct comparison with the subgroups of vestibular deafferented cats. GAD67: glutamic acid decarboxylase, the enzyme for GABA synthesis; D: day; IVN: inferior vestibular nucleus; LVN: lateral vestibular nucleus; MVN: medial vestibular nucleus; SVN: superior vestibular nucleus; TTX: tetrodoxin; UL: unilateral labyrinthectomy, UVN: unilateral vestibular neurectomy. *p<0.0001; +p<0.01 versus control. Scale bar: 90 *µ*m and *n = *4 animals per group.

**Figure 3:**
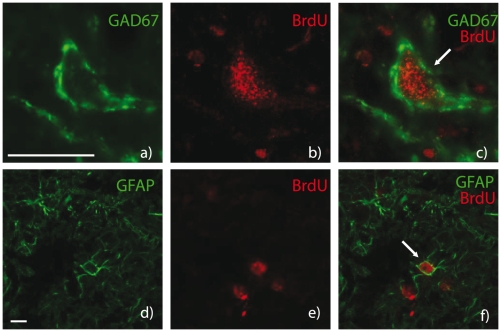
Newly generated cells survive and differentiate into GABAergic neurons and astrocytes exclusively after vestibular neurectomy. Confocal analysis of newly generated glutamate decarboxylase (GAD)-67-immunoreactive (Ir) neurons (**a, b, c**) and newly generated glial fibrillary acidic protein (GFAP)-immunoreactive (Ir) cells (**d, e, f**) in the deafferented MVN 60 days after UVN. BrdU was administered 3 days after the nerve section, and the animals were killed 57 days after. BrdU-immunoreactive cells are in red (**b, c, e, f**) and the other markers of cell differentiation in green: GAD67 (**a, c**) and GFAP (**d, f**). Scale bars represent 50 *µ*m (**a**) and 10 *µ*m (**e**).

Whatever the nature of vestibular deafferentation, the SVN did not exhibit GAD67-immunoreactive changes. After UVN, the pattern of GAD67^+^ expression enhanced in the deafferented MVN, IVN and LVN for all the postoperative delays. Compared to controls, the number of GAD67^+^ cells increased at D1, peaked at D3 in the LVN (+301.58% compared to control group), and at D7 in the MVN and the IVN (+328.57% and +458.26% respectively compared to control group) and then decreased slightly but still remained significantly higher than control and TTX groups. At D60 post-UVN, the number of GAD67^+^ cells remained significantly high (p<0.0001). Comparatively, UL led to a slighter increased number of GAD67^+^ cells at D1 with a peak of expression at D3 (+295.91% in the MVN, +256.09% in the IVN, +215.09% in the LVN compared to controls). At D7 the number of GAD67^+^ cells decreased slightly and remained significantly high at D60 (Figure 2). After TTX injection, the number of GAD67-immunoreactive cells increased slightly at D1, peaked at D3 (+161.63% in the MVN, +142% in the IVN and +115.62% in the LVN compared to controls) and declined progressively at D7 to reach control values at D30 for all the VN (Figure 2B). The double-immunohistochemistry against BrdU and GAD67 revealed numerous cells co-localizing these two markers in the UVN groups. So, a large subset of the newborn cells that incorporated BrdU at 3 days post-UVN, differentiated into GABAergic neurons and survived up to 2 months, as attested by confocal microscopy (Figure 3 a,b,c). Conversely, no colocalization of BrdU and GAD67 immunostainings was detected in the VN of both the TTX and the UL groups of cats at this chosen post-operative delay, suggesting a lack of newborn GAD67 neurons in these groups.

### GFAP expression in the deafferented vestibular nuclei

**Figure 4:**
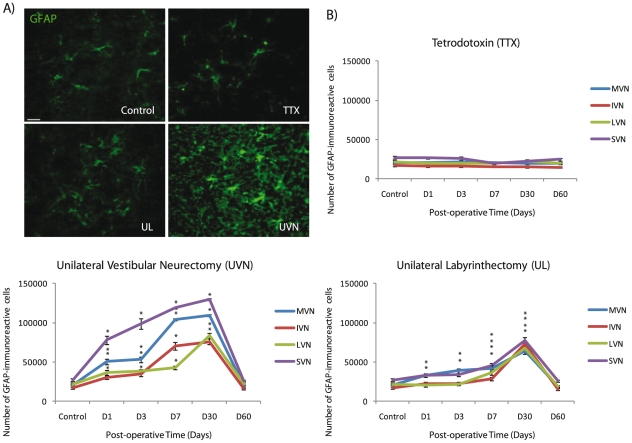
The more severe the deafferentation, the stronger the astroglial reaction. (**A**) The astroglial reaction in the vicinity of the lesion was stronger after vestibular nerve section than after unilateral labyrinthectomy or unilateral TTX injection. Illustration of GFAP immunoreactivity in the deafferented medial vestibular nucleus (MVN) in a representative control cat and in three experimental cats subjected to TTX, UL, and UVN and analyzed 30 days after these different kinds of vestibular deafferentations. (**B**) Quantitative evaluation of the effects of the different kinds of vestibular loss on GFAP-immunoreactive cells in the deafferented vestibular nuclei (VN). Data are mean values (± SEM) of the number of GFAP-immunoreactive cells in the deafferented vestibular nuclei of control and experimental groups. Only values recorded on the lesioned side are illustrated. Data from both sides of control cats were pooled for direct comparison with the subgroups of vestibular deafferented cats. GFAP: glial fibrillary acidic protein; D: day; IVN: inferior vestibular nucleus; LVN: lateral vestibular nucleus; MVN: medial vestibular nucleus; SVN: superior vestibular nucleus; TTX: tetrodoxin; UL: unilateral labyrinthectomy, UVN: unilateral vestibular neurectomy. * p<0.0001 versus control. Scale bar: 20 *µ*m and *n = *4 animals per group.

We found that UVN provided the strongest local astroglial reaction. In all the deafferented VN, the number of GFAP^+^ cells strongly increased from D1 to D30. Data recorded at D30 were significantly higher than in the control group (+443.15% in the MVN; +360.29% in the IVN; +287.66% in the LVN; +381.07 in the SVN; p<0.0001). At D60, however, the number of GFAP^+^ cells returned to control values. Note that among the VN, the deafferented SVN exhibited the highest number of GFAP^+^ cells whatever the post-UVN delays. Compared to the control group, we observed that after UL the number of GFAP^+^ cells increased significantly in all the deafferented VN from the first post-operative day (D1), peaked at D30 (+216.66% in the MVN, +341.28% in the IVN, +213.5% in the LVN, and +187.14% in the SVN compared to controls; p<0.0001) and then reached control values at D60 (Figure 4). TTX injection in the inner ear led nevertheless to a non-significant increase of the number of GFAP^+^ cells in the VN. Furthermore, since the newly generated cells of the UVN group were neurochemically characterized by double-immunofluorescence stainings, we found that among the numerous newborn cells that incorporated BrdU at D3, a subset differentiated into astrocytes at D60, colocalizing BrdU and GFAP markers (Figure 3 d,e,f). In contrast, no BrdU/GFAP double-immunostainings were observed in the VN at the chosen post-operative delay in the TTX and the UL groups, suggesting a lack of newly generated astrocytes after these two kinds of vestibular deafferentations.

### Behavioural deficits after vestibular deafferentation

In mammals, the leading ocular motor effect of vestibular deafferentation is a high frequency, mainly horizontal, spontaneous nystagmus, with its quick phase directed toward the intact side [23]. Lesioned animals also exhibit postural imbalance, yaw curvature of the spine with midscapular point and sacrum directed to the lesioned side, circling and rolling toward the lesioned side, extension of the contralateral forelimb, and head nystagmus [23]. After deafferentation, they adopt a largely increased support surface, which decreases over time. Then, when they try to move, they fall down toward the lesioned side. The deficits observed after a unilateral vestibular deafferentation were the same after TTX injection, UL, or UVN, but the time course of recovery differed according to the severity of the damage.

#### Spontaneous nystagmus

**Figure 5:**
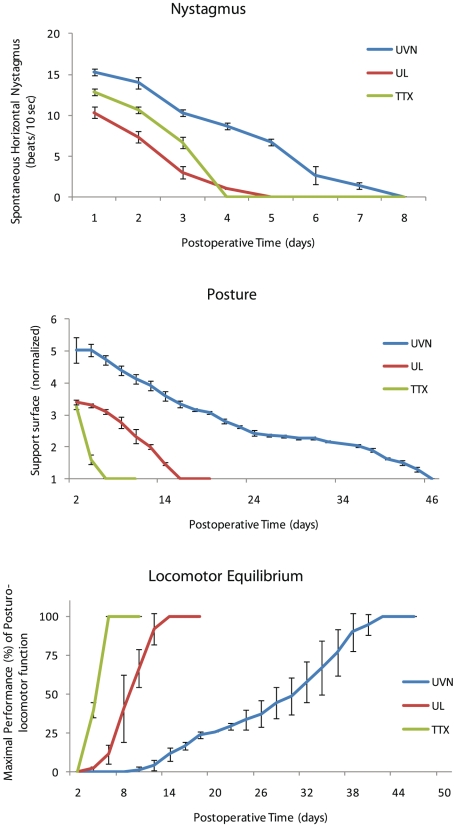
Behavioural recovery time course is governed by the nature of the vestibular damage. (**A**) Curves illustrating the time course (on the abscissae) of disappearance of horizontal spontaneous nystagmus (HSN) frequency (on the ordinates) for each group of vestibular deafferented cats at different postoperative days. Each data point represents the mean number of HSN quick phase movements in 10 s for 4 animals (five repeated measures per animal per sampling). Error bars represent SEM. (**B**) Curves indicating the mean postoperative recovery of the support surface in the three experimental groups of cats (TTX, UL, and UVN). Data recorded after vestibular deafferentation were related to individual references and normalized with respect to the preoperative values referred to unity (one being close to 50 cm^2^). Standard errors of the mean are reported as vertical lines. Note the delay in time recovery after UVN as compared to the other groups: 46 days instead of 6 for TTX and 16 for UL groups. **(C)** The maximal performance (Max P) is defined as the highest beam rotation speed that did not lead to a fall on four consecutive crossings. The curves are expressed in percent of the preoperative maximal performance (in the ordinates) as a function of the postoperative time in days (on the abscissae). Standard errors of the mean are reported as vertical lines.

**** Animals displayed a horizontal spontaneous nystagmus whatever the nature of vestibular deafferentation: TTX injection, UL, or UVN (Figure 5A). The compensation profile of the ocular nystagmus for the TTX and UL groups did not differ significantly: at the first day post-deafferentation (D1), the frequency of the spontaneous nystagmus was 9.5 and 10 beats/ 10 sec respectively. It decreased gradually over time and reached 1.5 and 3 beats/ 10 sec at the third post-deafferentation day, and then disappeared completely at the fourth day. In contrast, after UVN the time course of vestibular nystagmus compensation was delayed relative to the TTX and UL groups (p<0.0001). Nystagmus frequency was 15 beats/sec the first day following UVN and declined linearly until its total disappearance at the eighth day post-UVN.

#### Posture function recovery

In four-footed animals standing erect, vestibular syndrome leads to an increased support surface delimited by the four paw pads. This parameter provides a good estimation of postural in stability and recovery. It displays the tonic asymmetries of extensor and flexor muscles of the anterior and posterior paws induced by the vestibular deafferentation [10], [24], [25]. Return to control values recorded pre-operatively was faster for the TTX group (6 days) than for the UL group (16 days) or the UVN group (46 days) (p<0.0001) (Figure 5B). The variance analysis (ANOVA) established significant effects depending on the groups (p<0.0001), the postoperative time (p<0.0001), and the interaction between these two factors.

#### Locomotor balance recovery

As observed for the posture function, animals of the TTX group showed the fastest recovery of their dynamic locomotor balance. They were able to cross the rotating beam at their maximal performance (Max P) at the 6th day after deafferentation, whereas the cats of UL group reached their Max P 14 days after deafferentation (Figure 5C). In the UVN group, cats managed to walk on the rotating beam from 12–14 days after surgery and reached their Max P only 47 days after. Variance analysis of the locomotor balance function recovery (ANOVA) demonstrated significant effects depending on the groups (p<0.0001), the postoperative time (p<0.0001), and the interaction between these two factors (p<0.0001).

## Discussion

### Neurogenesis and astrogenesis responses in the vestibular nuclei: a structural process specific to the UVN model of vestibular deafferentation

We show for the first time that the VN become a neurogenic zone in response to specific vestibular damage: UVN led to a high number of newborn cells in the deafferented VN, as previously described in the same model [9]. These newly generated cells survived up to two months, differentiated into GABAergic neurons, astrocytes, or microglial cells, and further contributed to fine vestibular compensation [10]. By contrast, the UL model induced a functional loss of vestibular input, and did not show any BrdU^+^ cells in the VN complex. TTX unilateral injection in the inner ear which induces a transient and functional blockade of electric activity of the ipsilateral vestibular nerve did not lead to any cell proliferation in the VN at the different post-operative times analyzed. One possible hypothesis explaining the reactive cell proliferation in the VN after UVN could be that new cells would contribute to neuronal balance between apoptosis and neurogenesis, as in the subgranular zone. Nevertheless, it appears that apoptotic cells were not detected in VN after UVN (TUNEL method and caspase 3 immunostrainings, results not shown). Furthermore, after vestibular nerve transection, BrdU administration 3 days post-UVN labelled newborn cells that survived up to 2 months and differentiated into GABAergic neurons, astrocytes or microglial cells. Accordingly, such long-lived cells could not be considered as expiring cells and consequently, BrdU labeling would not be reliable to DNA repair or re-enter in abortive cell cycle of dying cells. Finally, The presence of nestin-Ir cells (a neural stem cell marker) in the deafferented VN after UVN [9] and the total blockade of cell proliferation in the VN in cats infused with an antimitotic drug [10] strongly suggests that the BrdU immunolabelling is related to mitotic activity instead of DNA repair or apoptosis events.

Few studies have investigated reactive cell proliferation existence in the vestibular nuclei following vestibular lesion. Campos-Torres have found an increase in BrdU^+^ cells after TTX unilateral injection or UL in rats. Nevertheless, these cells all differentiated into glial cells [6]. More recently, Zheng et al. observed also an increase of cell proliferation in the vestibular nucleus complex after bilateral vestibular deafferentation in the adult rat [26]. Approximately half of the newly generated cells population survived for up to 1 month in the experimental group. Intriguingly, they also found an increase of BrdU^+^ cells with a significant survival rate in the sham-operated group, suggesting that this reactive plastic event might not have a functional significance in rats. In this study however, the nature of the BrdU^+^ cells was not investigated: newborn surviving cells could so far differentiate into glial cells or, as suggested by authors, be a part of the microglial reaction already described after vestibular lesions and not necessary neurons [6]. The discrepancy with our previous data [10] could be related to the species differences and/or the nature of the surgeries: Zheng et al. practiced a bilateral aspiration of the contents of the canal ampullae and the utricle and saccule in rats, whereas we cut unilaterally the VIII^th^ cranial nerve in cats.

In the UVN cat model, the severity of the deafferentation –total and fast, structural and functional – could induce tissue modifications allowing the cellular microenvironment to switch from a non-neurogenic to a neurogenic area. This agrees with the idea that stem cell function is modulated according to physiological changes and needs [27]. Accordingly, the reactive cell proliferation observed in our model of UVN cats may be due in part to the high concentrations of pro- and or anti-inflammatory molecules released by the fast degeneration of the vestibular nerve. This hypothesis is supported by a recent study conducted in the rat model of total unilateral vestibular loss that clearly showed a strong inflammatory response [28]. It is known that according to the pathological conditions, inflammatory responses can participate in cell proliferation, migration, differentiation, survival, and incorporation of newborn cells into neural networks [29]. We believe that since anti-inflammatory cytokines have mainly pro-neurogenic effects [30], they may be major players influencing the neurogenic environment in our animal model of UVN. From the present data, it can be concluded that the nature and the severity of the vestibular injury could promotes specific tissue modifications enabling punctually the neurogenic potential in the deafferented VN, leading to reactive neurogenesis and afterwards functional integration of the newborn neurons [31].

### GAD67 expression in the VN: a common reaction whose intensity depends on the nature of the vestibular damage

Results showed first that the number of GAD67^+^ cells increased in the deafferented VN after vestibular damages, and second that GAD67 increased expression depended on the nature of the vestibular damage. Whatever the post-lesion days, we found that UVN induced stronger and longer GAD67 expression than UL. TTX injection triggered the weakest and the most transient GAD67 expression. The intense GAD67 expression observed after UVN comes in part from differentiation of the newly generated cells into GABAergic neurons and from pre-existing GABAergic neurons increasing the level of their synthesizing enzyme. Intriguingly, a strong increase in GABA-staining varicosities was also observed in the deafferented VN following UVN. This result suggests a trophic effect of GABA through activation of GABA_A_ receptors endowed by possible neural stem cells [32]. GABA is indeed known to provide differentiation of newborn cells into a neural lineage and to contribute to neurite outgrowth during development and adult neurogenesis [33].

The different GAD67 patterns of expression observed in the three experimental groups had duration-time profiles that coincided with the behavioural recovery time-courses observed in each model of vestibular lesion. GABA and its two receptors GABA_A_ and GABA_B_ are known to coordinate the vestibular pathways and to restore vestibular functions [34]. Injections or infusions of GABA_A_ antagonists accelerate postural recovery of vestibular deafferented animals, while GABA_A_ agonists have opposite effects [35], [36]. Interestingly, in accordance with results of Tighilet and Lacour [37] that observed a bilateral GAD67 increased expression in the VN one year after UVN, we also observed a bilateral GAD67 increased expression in the VN as soon as two months after UVN. At the behavioural level, vestibular compensation at this time was completed for the majority of vestibular symptoms. Since oculomotor function recovery is faster than posturo-locomotor recovery and depends on different plasticity mechanisms, we propose that this bilateral GAD67 increased expression would be a plastic neurochemical mechanism involved in the posturo-locomotor recovery and in its long-term maintenance. In line with this, chronic infusion of GABA_A_ receptor antagonists in the deafferented VN of vestibulo-lesioned guinea pigs modified the expression of postural symptoms, with no alteration of the oculomotor deficits [36]. Moreover, antimitotic drugs that block reactive cell proliferation after UVN drastically delay only the posturo-locomor functions and have no incidence on horizontal nystagmus [10]. Resting discharge rebalance in the VN probably involves a decrease in the efficacy of GABA_A_ and GABA_B_ receptors in the intact side and an increase in neuronal excitability on the damaged side [38], [39], [40]. These mechanisms agree with electrophysiological data showing a spontaneous resting activity imbalance between the homologous VN at the acute stage after vestibular damage, which is rebalanced at the compensatory stage in the alert guinea pig [41] and the cat [42]. Different neurochemical and electrophysiological events may have occurred in the VN of the TTX and the UL groups, which expressed different rates of GAD67 expression and faster vestibular function recovery than the UVN group. Other hypotheses can be proposed to explain the contribution of the GABAergic system to recovery of vestibular functions. As previously described [9], [10], newborn VN GABAergic neurons may integrate neural networks and contact different neuron targets in the VN to rebalance resting activity between the homologous VN. An alternative hypothesis is that the vestibular injury recapitulates developmental processes, leading to a change of expression of cation-chloride-cotransporters in the deafferented VN. Since cation-chloride cotransporters expression regulates the intracellular Cl^-^ concentration, when GABA targets its GABA_A_ receptor, it can lead to depolarizing effect in immature neurons by Cl^-^ extrusion to the cell [43], [44]. Both mechanisms would restore the resting activity on the deafferented VN, favouring vestibular compensation.

### GFAP expression in the VN: a non common response to vestibular damage

UVN provided an intense astroglial reaction in the deafferented VN from D1; subsequently, the increased expression of GFAP peaked at D30 and returned to control level at D60. Our results showed also that UL provided a weaker astroglial reaction peaking at D30 in the deafferented VN. Further, unilateral TTX injection did not modify GFAP expression in the VN whatever the time-delay observed. These findings agree with results in rats that showed a lack of astroglial reaction in the VN after transtympanic TTX injection, and an astroglial reaction after UL [6]. The authors suggested that contrary to TTX injection, UL-induced astroglial reaction would favour the expression of pro- and/or anti-inflammatory molecules like cytokines, known to regulate different steps of gliosis after central or peripheral injuries [45]. Hence, UVN induces more acute and faster nerve degeneration than UL, which could explain the more intense GFAP expression. In line with the hypothesis of Campos-Torres (2005), we suggest that UVN and consequently acute nerve section would recruit astrocytes and microglia to drive inflammatory response. Two months after UL and UVN, GFAP^+^ in the VN returned to control level, probably because the inflammatory process had stopped. As reviewed by Robel [46], our results are in line with the time course of the astroglial reaction observed after different kind of central nervous system damages. Following injury, astrocytes start to express histological and enzymatic changes such as increased GFAP intermediate filament synthesis and somatic hypertrophy, which depend on the characteristics and the intensity of the injury [47]. Since astrocytes are known to regulate ionic homeostasis for an optimal neural environment, they adapt themselves to the severity of lesion. In addition, Robel described that in severe injuries, some reactive astrocytes can proliferate and incorporate BrdU as we observed in the deafferented VN after UVN [46]. Likewise, the authors also indicate that within an injury site, reactive astrocytes can be a source of cells with stem cell potential. So after UVN, astrocytes located in the VN might be instructed by specific acute signals to enable neurogenic features allowing subsequently cell proliferation, neuronal survival, and differentiation of the newly generated cells.

### Behavioural correlates and clinical implications

The same acute behavioural syndrome was found in the three groups of cats after unilateral vestibular deafferentation either by TTX inner ear injection, UL, or UVN. The patterns of oculomotor and posturo-locomotor functional recovery differed nevertheless according to the groups, with the fastest recovery in the TTX group (1 week) than in the UL group (2 weeks) or the UVN group (6 weeks). This means that the nature of the vestibular deafferentation induces different time courses of functional recovery; the more severe and acute the deafferentation, the longer the duration of vestibular compensation. Indeed, the VN on the lesioned side are submitted after UVN to a fast, sudden and total deafferentation, both structural and functional (like surgery of Menière's disease, vestibular neuritis) [3], [48]. By contrast, when the sensory hair cells at the peripheral labyrinth are destroyed (UL), the VN do not show so fast and complete structural deafferentation, because the primary vestibular neurons in Scarpa's ganglion are still alive. The VN are only functionally deafferented and this process can be very progressive and slow, or faster, depending again on the aetiology. A sophisticated structural mechanisms such as neurogenesis is not required on this case for recovery. Increased expression of neurotransmitters, neurohormones, and/or neurotrophic factors is sufficient to avoid vestibular cell apoptosis and to accelerate functional recovery [3]. Finally, blockade of action potentials in the vestibular nerve by TTX, in a reversible way and for a short time, is followed by very fast recovery. Correlatively, only a weak and transient increased expression of GAD67 was observed. We propose that there is no need for the central nervous system to express robust plasticity mechanisms in such transient and/or reversible deficits.

In conclusion, we show for the first time *in vivo* that the vestibular nuclei can become a neurogenic zone in specific conditions. Depending on the nature of the vestibular damage, different and more or less specific cellular plasticity mechanisms are involved in the recovery process, and these recovery mechanisms underlie different recovery time courses. These new data are of interest for approaches to brain repair in understanding the factors favouring the expression of functional neurogenesis in adult mammals. Moreover, they are of clinical relevance in vestibular pathology for both drug treatment and rehabilitation in vestibular loss patients with different aetiologies.

## Materials and Methods

### Ethics Statement

All experiments were carried out in strict accordance with the National Institute of Health Guide for Care and Use of Laboratory Animals (NIH Publication n° 80–23) revised 1996 for the UK Animals (Scientific Procedures) Act 1986 and associated guidelines, or the Policy on Ethics approved by the Society for Neuroscience in November 1989, and amended in November 72 1993. No institutional review board or ethics committee are requested to approve animal research in France. The people performing the experiments on the animals are licensed (ML:A13055-25,01.1914; BT:A13055-75 25,13.363). Every attempt was made to minimize both the number and the suffering of animals used in this experiment. Cats were housed in a large confined space with normal diurnal light variations and free access to water and food.

### Surgeries

#### Unilateral Vestibular Neurectomy

Animals were anaesthetized with ketamine (20 mg/kg, *i.m*.; Rhône Poulenc, Mérieux, France), received analgesic (Tolfedine, 0.5 ml, i.m.; Vetoquinol, Lure, France) and were kept at physiological body temperature using a blanket. The vestibular nerve was sectioned on the left-side at the post-ganglion level in order to leave the auditory division intact after mastoidectomy, partial destruction of the bony labyrinth, and surgical exposure of the internal auditory canal [49]. Animals were maintained under antibiotics for 7 days and analgesics for 3 days. The classical postural, locomotor, and oculomotor deficits displayed by the animals in the days following nerve transection were used as criteria indicating the effectiveness of the vestibular nerve lesion. Completeness of vestibular nerve section had been assessed by histological procedures in previous studies [50].

#### Unilateral Labyrinthectomy

The aim of a unilateral labyrinthectomy (UL) was to remove all vestibular sensory regions. Surgical labyrinthectomy was practised with the same approach as for UVN. After ossicles were removed and their tensor muscles were withdrawn, the whole vestibular receptors in the inner ear cavity were destroyed with a diamond drill. The cavity was then closed up with spongel and skin was stitched up.

#### Unilateral tetrodotoxin (TXX) injection

TTX has been used to block action potentials transiently and to shut down the vestibular nerve [6], [51]. Surgery was carried out using an operative microscope according to the same approach described above. When the left tympanic bulla of animals showed round and oval windows, vestibular afferents were blocked with an injection within the windows of 150 µl of 3 mM TTX (Tocris, Cookson Ltd, Bristol, UK) diluted in a phosphate buffer (PB 0.1 M, pH 7.4). This TTX dose induced blockage of the nervous activity during 3 days.

### Study design

**Figure 6:**
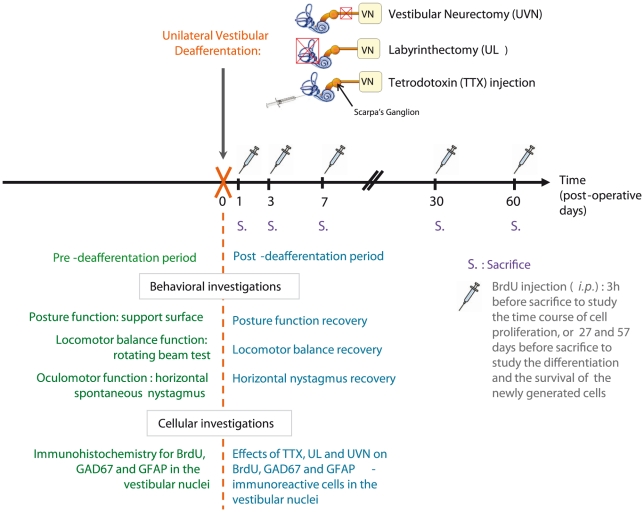
Study design. Experimental protocol elaborated for studying the effects of the three kinds of vestibular deafferentations (tetrodotoxin injection, unilateral labyrinthectomy, or unilateral vestibular neurectomy) on cell proliferation (BrdU marker), astrogenesis (GFAP marker), and GABA immunostaining (GAD67 marker) in the vestibular nuclei at different post-operative days. The effect of these three kinds of vestibular deafferentations on functional recovery was analyzed in all groups by oculomotor and posturo-locomotor tests. The UL, UVN, and TTX models reproduce gradual, sudden, and transient unilateral loss of vestibular function, respectively, and are intended to mimic different vestibular pathologies or diseases (see text). TTX: tetrodoxin; UL: unilateral labyrinthectomy, UVN: unilateral vestibular neurectomy; GAD67: glutamic acid decarboxylase the enzyme for GABA synthesis; GFAP: glial acidic fibrillary protein; BrdU: 5-bromo-2′deoxyuridine; *i.p.*: intraperitoneal.

To determine the effects of the different vestibular deafferentation models on both the plasticity mechanisms at a cellular level in the VN and on the time course of the cats' recovery at a behavioural level, we studied eight groups of animals. Based on our anterior data, to study the time course of reactive cell proliferation in the VN after UVN, we selected five post-deafferentation survival periods: 1, 3, 7, 30, and 60 days (D1, D3, D7, D30 and D60) (Figure 6). i) A group of sham-operated animals (n = 4) were used as a control group; they were submitted to anaesthesia and surgical approach of the different vestibular deafferentations without sectioning the nerve or destroying the receptors. These sham-opearted animals were killed at different survival periods: two at three days and two at one month. They all received an intraperitoneal injection of BrdU 3 hours before sacrifice. ii) animals that underwent a unilateral vestibular neurectomy (UVN group: total n = 20), sacrificed at D1 (n = 4), D3 (n = 4), D7 (n = 4), D30 (n = 4), and D60 (n = 4 these cats were also used for the behavioural study, since we had shown that the training course on the rotating beam did not trigger modifications of the cell proliferation in the VN [10]). iii) animals that underwent a unilateral labyrinthectomy (UL) (UL group: total n = 20) were sacrificed at the following postoperative days: D1 (n = 4), D3 (n = 4), D7 (n = 4), D30 (n = 4), and D60 (n = 4 for both the cellular and the behavioural studies). iv) animals that underwent a unilateral tetrodrotoxin (TTX) injection in the inner ear (TTX group: total n = 20) were sacrificed at different postoperative days for the cellular study: D1 (n = 4), D3 (n = 4), D7 (n = 4), D30 (n = 4), and D60 (n = 4 for both the cellular and the behavioural studies). To study the time course of reactive cell proliferation, whatever the experimental groups, animals belonging to the D1, D3, D7, D30, and D60 postoperative days received 5-bromo-2′-deoxyuridine (BrdU) 3 h before death in order to examine the presence of adult newborn cells in the VN. To study the survival and the differentiation of the proliferating cells in the VN, two additional groups of cats were submitted to UVN, injected with BrdU (i.p.) at D3 when the cell proliferation reached its peak, and killed 30 days (n = 4) and 60 days (n = 4) after UVN (Figure 6).

### Behavioural investigations

#### Spontaneous nystagmus recovery

For nystagmus recording, one day after surgery, the cat was placed on an apparatus with its head fixed, thus maintaining the horizontal semi-circular canals in the horizontal plane. The frequency of the HSN was measured in the light as the number of quick phase beats towards the contralateral side relative to UVN in 10 sec (five repeated measures per animal per sampling time) with a video camera (Sony HDV) [25].

#### Posture recovery

The support surface measure serves to evaluate the postural stability of the animal. Posture deficits and recovery were evaluated by measuring the surface delimited by the four legs of the cat while standing erect at rest, without walking. Support surface can be regarded as a good estimate of postural control because it reflects the cat's behavioural adaptation compensating the static vestibulospinal deficits induced by the vestibular lesion [*cf* 24]. As a rule, the surface was very small in the normal cat (about 50–100 cm^2^) and greatly increased in the days following unilateral vestibular lesion. To quantify the support surface, cats were placed in a device with a graduated transparent floor that allowed them to be photographed from underneath. Five repeated measurements were done for each cat tested at each postoperative time, and an average was calculated for each experimental session. The support surface was measured as the surface delimited by the four legs by an image analysis system (canvas, 9™, Deneba software, Miami, FL). Data recorded after vestibular lesion were compared to pre-lesion values by using individual references, that is, each animal acted as its own control.

#### Equilibrium function recovery

Locomotor balance function was quantified using the rotating beam experimental device [49]. Two compartments (0.5×0.6×0.5 m) were connected by a horizontal beam (length: 2 m; diameter: 0.12 m). The beam, placed 1.2 m off the ground, could be rotated along its longitudinal axis with a constant angular velocity ranging from 0° to 588.4 °/s (about 1.5 turn/s). Behavioural training on the rotating beam consisted in depriving the animals of food for 12 h before the first training session. Animals were conditioned to cross over the beam and were rewarded by a small piece of fish (or meat) placed in a small bowl in the target compartment. First crossings were made on the immobile beam and, thereafter, on the rotating beam. As a rule, rotation velocity of the beam was progressively increased after four consecutive trials without fall. Equilibrium function was thus quantified by measuring the highest speed of beam rotation that did not induce a fall. This maximal rotation speed determined the maximal locomotor balance performance (Max. P.). Preoperative training on the rotating beam necessitated 6 to 10 training periods of 1 h per day, depending on the cats. Training was stopped when the cats' Max. P. was reached and stabilised at its highest level, which was found to be remarkably similar from one cat to another.

#### Statistical analysis

Statistical analysis consisted of an analysis of variance (ANOVA) to test for changes at the different post-lesion delays in the spontaneous nystagmus, the support surface, and the maximal equilibrium performance of the cats. Results were considered significant at p<0.05. (Table 1).

**Table 1:** Statistical analysis of the effects of three vestibular deafferentations on vestibular compensation at the behavioural level.

Source of variation	df	F	P
**Horizontal nystagmus**			
Group (UVN/UL/TTX)	2	372.93	0.0001*
Post operative time	9	982.15	0.0001*
Group×post operative time	18	74.05	0.0001*
**Posture**			
Group (UVN/UL/TTX)	2	1197.83	0.0001*
Post operative time	24	720.20	0.0001*
Group×post operative time	48	145.99	0.0001*
**Equilibrium function**			
Group (UVN/UL/TTX)	2	262.50	0.0001*
Post operative time	21	203.04	0.0001*
Group×post operative time	42	43.59	0.0001*

Repeated-measure analysis of variance of the horizontal spontaneous nystagmus, posture recovery, and equilibrium function recovery. Group (unilateral vestibular neurectomized cats versus unilateral vestibular labyrinthectomized cats versus tetrodotoxine cats), and survival period are the main fixed effects providing the sources of variation among cats, as also illustrated by the significant interaction between these two variables (*).

df: degree of freedom; F: Scheffé's test; P: Probability level.

### Cellular investigations

#### Tissue preparation

BrdU (10 mg/ml, Sigma, Saint Quentin Fallavier, France) was dissolved in a solution of sodium chloride (NaCl) 0.9% heated to 56°C and injected in animals (200 mg/kg). Cameron and McKay (2001) showed in adult rat dentate gyrus that a single dose of BrdU 100, 50 or 25 mg/kg (body weight, *i.p*.) labeled 60%, 45% and 8% of S-phase cells respectively [52]. At 300 mg/kg, BrdU labeled most S-phase cells and had no physiological side effects. So, in line with conclusions of Taupin (2007), 200 mg/kg is a saturating concentration of BrdU for studying adult neurogenesis [53]. BrdU doses were not likely to generate side effects, but were sufficient to mark the cells in S-phase synthesizing DNA. Cats of each group were deeply anaesthetised with ketamine dihydrochoride (20 mg/kg, *i.m*., Merial, Lyon, France) and killed by paraformaldehyde perfusion 3 hours, 27 days, or 57 days after the BrdU injection according to their experimental group. After removal from the skull, brains were cut into several blocks containing the VN. The blocks were rapidly frozen with CO_2_ gas and stored at −80°C. Coronal sections (40-*µ*m-thick) were cut in a cryostat (Leica, Reuil-Malmaison, France) for immunochemistry.

#### Immunochemistry

Immunochemical labelling of BrdU-immunoreative (Ir) cells was performed according to previously validated protocols [9], [54]. For BrdU-immunostainings, free-floating sections were first rinsed in 0.1 M PBS and incubated with 2N HCl and 0.5% Triton-X100 in PBS (30 min, 37°C) for DNA hydrolysis. Then sections were rinsed in 0.1 M sodium tetraborate buffer, pH 8.5 before overnight incubation with the primary antibody at 4°C, followed by incubation with the secondary antibody for 1.5 h at room temperature, and visualised using horseradish peroxidase avidin-D (Vector). GFAP and GAD67 immunoreactivity assays were performed according to Tighilet, Brezun et al. [9]. After several rinses, sections were mounted on gelatin-coated slides, dehydrated, and cover-slipped in Depex mounting medium for peroxidase staining. Double-immunofluorescence stained sections were incubated with GFAP or GAD67 combined with BrdU^+^ protocol. The optimal antibody dilutions and staining procedures are described in Table 2. Differentiation of the newly generated cells was analysed with double-labelling analysis performed using confocal imaging with a Leica TCS SP2 laser scanning microscope equipped with a 63x/1.32 NA oil immersion lens. The fields of view were then examined by confocal microscopy, and 1-*µ*m-step Z series were obtained.

**Table 2:** Antibodies and methods of detection.

Marker	Primary antibody	Secondary antibody	Technique – coloration
BrdU	Mouse 1/100, Dako	Horse anti-mouse 1/200, Vector	DAB - brown
GFAP	Rabbit 1/200, Dako	Goat anti-rabbit 1/200, Vector	DAB - brown
GAD67	Mouse 1/10000, Chemicon	Horse anti-mouse 1/200, Vector	DAB - brown
BrdU	Rat 1/100, Oxford Biot	Rabbit anti-rat 1/200, Interchim	Alexa Fluor 594 - red
GFAP	Rabbit 1/200, Dako	Goat anti-rabbit 1/200, Interchim	AlexaFluor 488 - green
GAD67	Mouse 1/100, Chemicon	Rabbit anti-mouse 1/200, Interchim	Alexa Fluor 488 - green

Combination and sequential processing of primary and secondary antibodies used for immunohistochemical and dual immunofluorescent stainings for BrdU, GFAP, or GAD67.

#### Cell counts and statistical analysis

Cell counts were performed according to a previously validated protocol [10]. Great care was taken not to count blood cells as BrdU^+^ cells. The VN were identified through Berman's stereotaxic atlas. BrdU^+^, GFAP^+^, and GAD67^+^ were quantified for each VN (medial, inferior, superior, and lateral vestibular nuclei: MVN, IVN, SVN, and LVN, respectively) from selected serial frontal sections collected from the dorsal (5.2) to the caudal (12.1) part of the brainstem and depending on the size and on the rostrocaudal length of each VN. GFAP^+^ and BrdU^+^ cells, and GAD67^+^ neurons were analyzed in each VN on both sides (left/right: sham-operated cats; ipsilateral/contralateral: UVN-lesioned cats, see supporting informations). We decided to analyze the VN subdivisions instead of analyzing the whole set of nuclei because i) these markers are expressed differentially in the VN after UVN. ii) the VN are involved differentially in the vestibular compensation processes. The SVN, which is the structure mostly involved in oculomotor function, does not exhibit neurogenesis. Conversely, a large number of newborn neurons were observed in the MVN, LVN, and IVN, which are mainly associated with static and dynamic postural functions.

Because astrocytes immunostain for the GABA synthesizing enzyme GAD67 [55], we followed strict morphologic criteria to distinguish GAD positive neurons from astrocytes. While it is usually straight forward to distinguish large- and middle-sized neurons from glial cells, the distinction between small neurons and large glial cells can be challenging. The following criteria were used as characteristic for neurons: a centrally located nucleolus, a distinctive nucleus, visible cytoplasm, presence of dendritic processes, and larger cell body size. Glial cells were identified by the following criteria: sparse cytoplasm, and smaller cell body size [56].

The cell count was done with a Nikon microscope (Eclipse 80 i) equipped with a motorized X-Y-Z sensitive stage and a video camera connected to a computerized image analysis system (Mercator; Explora Nova, La Rochelle, France). The total number of immunolabelled cells was estimated using the optical fractionator method [57]. BrdU^+^, GFAP^+^, and GAD67^+^ were counted in optical disectors and sampled according to the so-called fractionator principles. The optical fractionator is a combination of the optical disector, a threedimensional probe used for counting, and fractionator sampling, a scheme involving the probing of a known fraction of the tissue [57]. Three sampling fractions are used with the optical fractionator method. First, the section sampling fraction (ssf) represents the proportion of microscopical sections of the entire, serially sectioned brain structure that is sampled for evaluation. The area sampling fraction (asf) corresponds to the proportion of the sectional area that is investigated within the sampled sections. And finally, the thickness sampling fraction (tsf) captures the part of the investigated cross-sectional area of the sampled sections. The estimated total number of particles (*N*) of a brain structure in an animal is obtained by multiplying the reciprocals of the fractions with the total particle count (∑*Q* ^−^) per brain structure, obtained with the optical disectors [58]: N =  ∑*Q* ^−^ (1/ssf)(1/asf)(1/tsf). The size of the region of interest (ROI) is implicitly determined from the combination of these fractions. This means that, with the optical fractionator technique, no information on the size of the ROI or the magnification of the microscope is needed. This also implies that this counting technique is independent of e.g. swelling and/or shrinking of the tissue during processing [59], [60]. For each labeling, the quantified sections were systematically selected with a size step of 480 *µ*m along the anteroposterior axis. We counted only Ir-cells in focus within the height of the dissector (10 *µ*m) and inside the limits of the counting frame without touching the forbidden lines [10]. Accordingly, the statistical analyses were evaluated by ANOVA to test the effects of the group (Control, TTX, UL or UVN), the side (deafferented vs intact), and the structure (MVN, IVN, LVN, SVN) on BrdU-, GFAP- and GAD67-positive cells and to determine whether there were any interactions between these variables. ANOVA was followed by *post-hoc* analysis with the Scheffé test (StatView II, SAS software Inc., Cary, NC) (Table 3). The coefficient of error (CE) of the estimated number of BrdU^+^, GAD67^+^ and GFAP^+^ cells in the ipsilateral and the contralateral VN of the sham and the experimental groups for each survival period tested is given in Tables S1, S2 and S3 in the supporting information. The CE values were within acceptable ranges as described in an earlier study [57].

**Table 3:** Statistical analysis of the effects of the three vestibular deafferentations on the BrdU, GFAP and GAD immunolabellings at the cellular level.

Source of variation	df	F	P
**BrdU positive cells**			
Group (Controls/UVN/UL/TTX)	3	394.67	0.0001*
Vestibular nuclei	3	5.87	0.0005*
Group×Vestibular nuclei	9	8.08	0.0001*
Side	1	247.13	0.0001*
Group×Side	3	400.71	0.0001*
Side×Vestibular nuclei	3	5.04	0.001*
Group×Side×Vestibular nuclei	9	8.10	0.0001*
**GAD67 positive cells**			
Group (Controls/UVN/UL/TTX)	3	2214.97	0.0001*
Vestibular nuclei	3	1707.44	0.0001*
Group×Vestibular nuclei	9	242.43	0.0001*
Side	1	10827.04	0.0001*
Group×Side	3	2375.57	0.0001*
Side×Vestibular nuclei	3	1296.03	0.0001*
Group×Side×Vestibular nuclei	9	289.97	0.0001*
**GFAP positive cells**			
Group (Controls/UVN/UL/TTX)	3	1689.38	0.0001*
Vestibular nuclei	3	432.71	0.0001*
Group×Vestibular nuclei	9	94.03	0.0001*
Side	1	2285.80	0.0001*
Group×Side	3	1637.98	0.0001*
Side×Vestibular nuclei	3	70.48	0.0001*
Group×Side×Vestibular nuclei	9	89.48	0.0001*

Repeated-measure analysis of variance of the number of BrdU-, GFAP- and GAD67-positive cells in the vestibular nuclei. Group (unilateral vestibular neurectomized cats versus unilateral vestibular labyrinthectomized cats versus tetrodotoxine cats), vestibular nuclei (MVN, medial vestibular nuclei; IVN, inferior vestibular nuclei; LVN, lateral vestibular nuclei and SVN, superior vestibular nuclei) and side (intact versus deafferented) are the main fixed effects providing the sources of variation among cats (*).

df: degree of freedom; F: Scheffé's test; P: Probability level.

## Supporting Information

Table S1 **Mean total BrdU immuno-positive cell numbers and CE of stereological analysis for estimation of total BrdU immuno-positive cells in the ipsilateral and contralateral vestibular nuclei complexes of the sham and the experimental groups of cats for each survival period tested.** Values are mean ± SEM; CE: coefficient of error, BrdU: 5-bromo-2′deoxyuridine; D: day; IVN: inferior vestibular nucleus; LVN: lateral vestibular nucleus; MVN: medial vestibular nucleus; SVN: superior vestibular nucleus; TTX: tetrodoxin; UL: unilateral labyrinthectomy, UVN: unilateral vestibular neurectomy. * indicates a significant difference (p<0.0001) between the ipsilateral and contralateral sides.
(PDF)

Table S2 **Mean total GAD67 immuno-positive cell numbers and CE of stereological analysis for estimation of total GAD67 immuno-positive cells in the ipsilateral and contralateral vestibular nuclei complexes of the sham and the experimental groups of cats for each survival period tested.** Values are mean ± SEM; CE: coefficient of error, GAD67: glutamic acid decarboxylase, the enzyme for GABA synthesis; D: day; IVN: inferior vestibular nucleus; LVN: lateral vestibular nucleus; MVN: medial vestibular nucleus; SVN: superior vestibular nucleus; TTX: tetrodoxin; UL: unilateral labyrinthectomy, UVN: unilateral vestibular neurectomy.
(PDF)

Table S3 **Mean total GFAP immuno-positive cell numbers and CE of stereological analysis for estimation of total GFAP immuno-positive cells in the ipsilateral and contralateral vestibular nuclei complexes of the sham and the experimental groups of cats for each survival period tested.** Values are mean ± SEM; CE: coefficient of error, GFAP: glial fibrillary acidic protein, the enzyme for GABA synthesis; D: day; IVN: inferior vestibular nucleus; LVN: lateral vestibular nucleus; MVN: medial vestibular nucleus; SVN: superior vestibular nucleus; TTX: tetrodoxin; UL: unilateral labyrinthectomy, UVN: unilateral vestibular neurectomy.
(PDF)
