# Supplementary material for: Neurogenic Potential of the Vestibular Nuclei and Behavioural Recovery Time Course in the Adult Cat Are Governed by the Nature of the Vestibular Damage
Source: PLoS One. 2011 Aug 11;6(8):e22262. doi: 10.1371/journal.pone.0022262 (PMC3154899; doi:10.1371/journal.pone.0022262)
Supplement: Table S1 — Mean total BrdU immuno-positive cell numbers and CE of stereological analysis for estimation of total BrdU immuno-positive cells in the ipsilateral and contralateral vestibular nuclei complexes of the sham and the experimental groups of cats for each survival period tested. Values are mean ± SEM; CE: coefficient of error, BrdU: 5-bromo-2′deoxyuridine; D: day; IVN: inferior vestibular nucleus; LVN: lateral vestibular nucleus; MVN: medial vestibular nucleus; SVN: superior vestibular nucleus; TTX: tetrodoxin; UL: unilateral labyrinthectomy, UVN: unilateral vestibular neurectomy. * indicates a significant difference (p<0.0001) between the ipsilateral and contralateral sides. (PDF) [file pone.0022262.s001.pdf]

	Sham-operated			
	ipsi		*contra*	
	mean ± sem	*CE*	mean ± sem	CE
MVN	20,1 ± 1,5	0,07	20,0 ± 1,7	0,08
IVN	30,6 ± 1,34	0.04	33,6 ± 1,8	0,05
LVN	23,1 ± 2,0	0,08	22,5 ± 1,9	0,08
SVN	23,8 ± 2,1	0,08	23,6 ± 1,86	0,07

	D1				D3				D7				D30			
	ipsi		*contra*		*ipsi*		*contra*		*ipsi*		*contra*		*ipsi*		*contra*	
	mean ± sem	*CE*	mean ± sem	CE	mean ± sem	CE	mean ± sem	CE	mean ± sem	CE	mean ± sem	CE	mean ± sem	CE	mean ± sem	CE
**TTX**																
MVN	16,0 ± 1,4	0,08	15,6 ± 1,2	0,07	16,6 ± 1,3	0,07	17,8 ± 1,3	0,07	14,1 ± 1,2	0,08	15,0 ± 1,4	0,09	15,6 ± 1,3	0,08	14,5 ± 0,9	0,06
IVN	28,2 ± 1,34	0,04	29,0 ± 1,3	0,04	25,6 ± 1,6	0,06	25,3 ± 1,8	0,07	24,9 ±1,6	0,06	23,9 ± 1,6	0,06	28,8 ± 2,1	0,07	25,1 ± 1,2	0,04
LVN	21,1 ± 1,5	0,07	19,5 ±1,7	0,08	20,6 ± 1,6	0,07	18,6 ± 1,3	0,06	20,9 ± 1,8	0,08	18,7 ± 1,4	0,07	18,9 ± 1,6	0,08	16,3 ± 1,2	0,07
SVN	17,2 ± 1,2	0,06	18,3 ± 1,4	0,07	17,2 ± 1,7	0,08	18,4 ±1,3	0,07	15,8 ± 1,4	0,08	16,3 ± 1,2	0,07	18,0 ± 1,1	0,06	17,9 ± 0,7	0,03
**UL**																
MVN	12,4 ± 1,0	0,08	13,4 ± 1,2	0,08	12,4 ± 1,2	0,09	13,4 ± 1,1	0,08	11,4 ± 0,9	0,07	12,0 ± 1,1	0,09	12,0 ± 1,1	0,09	13,2 ± 1,3	0,09
IVN	25,1 ± 1,8	0,07	23,1 ± 1,7	0,07	25,9 ± 1,7	0,06	23,7 ± 1,0	0,04	25,5 ± 1,7	0,06	23,7 ± 1,4	0,05	24,6 ± 1,2	0,04	22,4 ± 1,5	0,06
LVN	14,7 ± 1,3	0,08	13,2 ± 1,0	0,07	14,9 ± 1,1	0,07	13,9 ± 1,3	0,09	15,1 ± 1,4	0,09	16,5 ± 1,1	0,06	15,1 ± 1,3	0,08	13,1 ± 1,1	0,08
SVN	11,1 ± 0,9	0,08	12,3 ± 1,2	0,09	12,8 ± 1,2	0,09	13,7 ± 1,1	0,08	12,0 ± 1,1	0,09	11,6 ± 1,1	0,09	12,6 ± 1,1	0,08	14,9 ± 1,4	0,09
**UVN**																
MVN	101,3 ± 8,2 *	0,08	17,6 ± 1,5	0,08	1160,1 ± 81,7 *	0,07	15,7 ± 1,1	0,07	160,1 ± 13,7 *	0,08	15,7 ± 1,1	0,07	19,3 ± 1,9	0,09	18,9 ± 1,7	0,08
IVN	72,4 ± 6,1 *	0,08	17,5 ± 1,1	0,06	1746,0 ± 99,2 *	0,05	15,2 ± 0,9	0,05	153,0 ± 12,2 *	0,07	17,7 ± 1,3	0,07	15,9 ± 0,8	0,05	16,9 ± 1,1	0,06
LVN	132,3 ± 11,6 *	0,08	18,6 ± 1,4	0,07	2457,2 ± 117,2 *	0,04	17,7 ± 1,1	0,06	189,2 ± 14,4 *	0,07	20,4 ± 1,4	0,06	13,8 ± 1,3	0,09	13,2 ± 1,1	0,08
SVN	180,5 ± 15,5 *	0,08	14,5 ± 1,2	0,08	1561,7 ± 91,9 *	0,05	16,9 ± 1,0	0,05	121,1 ± 11,9 *	0,09	11,9 ± 1,0	0,08	14,9 ± 1,1	0,07	15,5 ± 1,0	0,06

Table 4. Mean total BrdU immuno-positive cell numbers and CE of stereological analysis for estimation of total BrdU immuno-positive cells in the ipsilateral and contralateral vestibular nuclei complexes of the sham and the experimental groups of cats for each survival period tested . Values are mean ± SEM; CE: coefficient of error, BrdU: 5-bromo-2'deoxyuridine; D: day; IVN: inferior vestibular nucleus; LVN: lateral vestibular nucleus; MVN: medial vestibular nucleus; SVN: superior vestibular nucleus; TTX: tetrodoxin; UL: unilateral labyrinthectomy, UVN: unilateral vestibular neurectomy. * indicates a significant difference ($p < 0.0001$) between the ipsilateral and contralateral sides.
