# Supplementary material for: Neurogenic Potential of the Vestibular Nuclei and Behavioural Recovery Time Course in the Adult Cat Are Governed by the Nature of the Vestibular Damage
Source: PLoS One. 2011 Aug 11;6(8):e22262. doi: 10.1371/journal.pone.0022262 (PMC3154899; doi:10.1371/journal.pone.0022262)
Supplement: Table S2 — Mean total GAD67 immuno-positive cell numbers and CE of stereological analysis for estimation of total GAD67 immuno-positive cells in the ipsilateral and contralateral vestibular nuclei complexes of the sham and the experimental groups of cats for each survival period tested. Values are mean ± SEM; CE: coefficient of error, GAD67: glutamic acid decarboxylase, the enzyme for GABA synthesis; D: day; IVN: inferior vestibular nucleus; LVN: lateral vestibular nucleus; MVN: medial vestibular nucleus; SVN: superior vestibular nucleus; TTX: tetrodoxin; UL: unilateral labyrinthectomy, UVN: unilateral vestibular neurectomy. (PDF) [file pone.0022262.s002.pdf]

	Sham-operated			
	ipsi		*contra*	
	mean ± sem	*CE*	mean ± sem	CE
MVN	98 ± 8,3	0,08	95,6 ± 8,2	0,08
IVN	73,8 ± 6,1	0,08	76,8 ± 5,7	0,07
LVN	111,3 ± 9,0	0,08	107,8 ± 8,8	0,08
SVN	64 ± 5,20	0,08	62,8 ± 4,0	0,06

	D1				D3				D7				D30				D60			
	ipsi		*contra*		*ipsi*		*contra*		*ipsi*		*contra*		*ipsi*		*contra*		*ipsi*		*contra*	
	mean ± sem	*CE*	mean ± sem	CE	mean ± sem	CE	mean ± sem	CE	mean ± sem	CE	mean ± sem	CE	mean ± sem	CE	mean ± sem	CE	mean ± sem	CE	mean ± sem	CE
**TTX**																				
MVN	151,1 ± 10,1 $^{+}$	0,06	84,4 ± 7,1	0,08	256,4 ± 17,5 *	0,06	75,8 ± 6,5	0,08	203,3 ± 12,1 *	0,05	63,3 ± 4,1	0,06	114,5 ± 8,6 $^{+}$	0,07	66,3 ± 5,3	0,08	100,0 ± 8,6	0,08	82,1 ± 7,2	0,08
IVN	79,2 ± 6,2	0,07	61,2 ± 4,6	0,07	178,6 ± 15,6 *	0,08	70,0 ± 6,0	0,08	118,8 ± 10,1 $^{+}$	0,08	77,6 ± 5,6	0,07	57,6 ± 4,8	0,08	57,6 ± 4,6	0,07	72,0 ± 6,0	0,08	58,1 ± 4,7	0,08
LVN	140,9 ± 11,5 $^{+}$	0,08	76 ± 6,0	0,07	244,3 ± 19,3 *	0,07	73,2 ± 6,1	0,08	205,8 ± 13,7 *	0,06	61,4 ± 3,8	0,06	98,9 ± 7,6	0,07	62,7 ± 3,9	0,06	100,8 ± 8,2	0,08	65,1 ± 4,9	0,07
SVN	80 ± 7,9	0,09	84,4 ± 7,7	0,09	84,0 ± 6,1	0,07	88 ± 7,3	0,08	66,0 ± 3,7	0,05	62 ± 2,0	0,03	62,3 ± 4,7	0,07	61,5 ± 4,6	0,07	60,0 ± 5,1	0,08	62,4 ± 5,0	0,08
**UL**																				
MVN	295,5 ± 17,3 *	0,05	77,4 ± 4,2	0,05	388 ± 12,8 *	0,03	88 ± 5,5	0,06	311,1 ± 17,1 *	0,05	73,3 ± 5,5	0,07	286,6 ± 12,4 *	0,04	67,5 ± 4,2	0,06	160 ± 8,7 *	0,05	57,4 ± 2,8	0,04
IVN	189 ± 10,8 *	0,05	73,5 ± 4,4	0,05	262,8 ± 11,7 *	0,04	68 ± 4,4	0,06	247,5 ± 13,3 *	0,05	72,5 ± 5,0	0,06	226,2 ± 10,0 *	0,04	68,8 ± 4,0	0,05	171 ± 9,6 *	0,05	64,4 ± 4,0	0,06
LVN	287,9 ± 15,6 *	0,05	71,9 ± 5,0	0,06	357 ± 13,9 *	0,03	75,8 ± 5,0	0,06	315 ± 19,6 *	0,06	64,5 ± 4,1	0,06	309,75 ± 16,9 *	0,05	66,7± 4,3	0,06	191,4 ± 10,2 *	0,05	62,4 ± 4,3	0,06
SVN	66 ± 4,3	0,06	80,4 ± 4,7	0,05	68.4 ± 4,0	0,06	64 ± 4,5	0,07	67 ± 4,0	0,05	62,4 ± 4,9	0,07	66,6 ± 4,0	0,06	63,1 ± 3,2	0,05	40 ± 3,2	0,08	43,1 ± 3,0	0,06
**UVN**																				
MVN	310,9 ± 15,4 *	0,04	76,36 ± 5,2	0,06	393,3 ± 19,8 *	0,05	73,3 ± 4,0	0,05	420 ± 20,1 *	0,04	64,5 ± 3,6	0,05	339,6 ± 20,3 *	0,05	77,7 ± 5,6	0,07	298,2 ± 15,2 *	0,05	72,3 ± 5,3	0,07
IVN	303,7 ± 16,1 *	0,05	70,2 ± 3,0	0,04	360,0 ± 17,3 *	0,04	67,5 ± 3,3	0,04	412,2 ± 24,5 *	0,05	55,2 ± 2,7	0,04	221,8 ± 12,8 *	0,05	63 ± 4,4	0,06	205,0 ± 12,1 *	0,05	68,4 ± 4,5	0,06
LVN	300,01 ± 13,0 *	0,04	66,01 ± 3,1	0,04	455,0 ± 19,5 *	0,04	66,5 ± 4,1	0,06	432,6 ± 26,1 *	0,06	77,8 ± 5,9	0,07	220 ± 12,6 *	0,05	80,8 ± 5,7	0,07	185,7 ± 6,8 *	0,03	61,5 ± 4,1	0,06
SVN	74 ± 5,1	0,06	78.1 ± 5,1	0,06	81,0 ± 5,1	0,06	80,4 ± 3,9	0,06	74 ± 3,3	0,04	76 ± 5,4	0,07	80 ± 6,1	0,07	83,4 ± 4,1	0,06	70 ± 4,7	0,06	73,0 ± 3,4	0,07

Table 5. Mean total GAD67 immuno-positive cell numbers and CE of stereological analysis for estimation of total GAD67 immuno-positive cells in the ipsilateral and contralateral vestibular nuclei complexes of the sham and the experimental groups of cats for each survival period tested. Values are mean ± SEM; CE: coefficient of error, GAD67: glutamic acid decarboxylase, the enzyme for GABA synthesis; D: day; IVN: inferior vestibular nucleus; LVN: lateral vestibular nucleus; MVN: medial vestibular nucleus; SVN: superior vestibular nucleus; TTX: tetrodoxin; UL: unilateral labyrinthectomy, UVN: unilateral vestibular neurectomy.
