# Supplementary material for: Neurogenic Potential of the Vestibular Nuclei and Behavioural Recovery Time Course in the Adult Cat Are Governed by the Nature of the Vestibular Damage
Source: PLoS One. 2011 Aug 11;6(8):e22262. doi: 10.1371/journal.pone.0022262 (PMC3154899; doi:10.1371/journal.pone.0022262)
Supplement: Table S3 — Mean total GFAP immuno-positive cell numbers and CE of stereological analysis for estimation of total GFAP immuno-positive cells in the ipsilateral and contralateral vestibular nuclei complexes of the sham and the experimental groups of cats for each survival period tested. Values are mean ± SEM; CE: coefficient of error, GFAP: glial fibrillary acidic protein, the enzyme for GABA synthesis; D: day; IVN: inferior vestibular nucleus; LVN: lateral vestibular nucleus; MVN: medial vestibular nucleus; SVN: superior vestibular nucleus; TTX: tetrodoxin; UL: unilateral labyrinthectomy, UVN: unilateral vestibular neurectomy. (PDF) [file pone.0022262.s003.pdf]

	Sham-operated			
	ipsi		*contra*	
	mean ± sem	*CE*	mean ± sem	CE
MVN	20160 ± 1595,29	0,07	19679 ± 1441,4	0,07
IVN	16520 ± 1371,4	0,08	15723 ± 1279,0	0,08
LVN	21462 ± 1880,62	0,08	20665 ± 1786,8	0,08
SVN	27080 ± 1987,5	0,07	26721 ± 1956,8	0,07

	D1				D3				D7				D30				D60			
	ipsi		*contra*		*ipsi*		*contra*		*ipsi*		*contra*		*ipsi*		*contra*		*ipsi*		*contra*	
	mean ± sem	*CE*	mean ± sem	CE	mean ± sem	CE	mean ± sem	CE	mean ± sem	CE	mean ± sem	CE	mean ± sem	CE	mean ± sem	CE	mean ± sem	CE	mean ± sem	CE
**TTX**																				
MVN	20679,9 ± 889,2	0,04	19880,0 ± 1004,9	0,05	21440,0 ± 1503,3	0,07	21200,04 ± 1664,19	0,07	20280 ± 1788,22	0,08	19660 ± 1412,48	0,07	19440 ± 1224,9	0,06	19498 ± 1131,8	0,05	19868 ± 1209,4	0,06	19671 ± 1278,7	0,06
IVN	15911,9 ± 1235,3	0.07	16936,0 ± 1230,1	0.07	15727,9 ± 949,7	0,06	15441,36 ± 987,83	0,06	15476 ± 913,83	0,05	14446 ± 947,33	0,06	14692 ± 969,5	0,06	14545 ± 1019,2	0,07	14456 ± 1113,6	0,07	15564 ± 1071,0	0,06
LVN	19716,0 ± 1572,20	0.07	19064,0 ± 1464,7	0,07	19656,0 ± 1454,7	0.07	20292 ± 1336,43	0,06	19396 ± 1412,72	0,07	20174 ± 1086,76	0,05	21868 ± 1232,6	0,05	20646 ± 1211,9	0,05	19864 ± 1340,6	0,06	19454 ± 1352,8	0,06
SVN	26720 ± 1512,5	0,05	26265,0 ± 1252,5	0.04	25621 ± 1946,8	0.07	24401 ± 1956,8	0,08	24160 ± 1647,5	0,06	25576 ± 1621,89	0,06	22600 ± 1392,5	0,06	23687 ± 1271,9	0,05	24456 ± 1426,0	0,05	23784 ± 1129,7	0,04
**UL**																				
MVN	33120 ± 2300,35 *	0,06	20060 ± 1733,38	0,08	38790 ± 2449,47 *	0,06	19230 ± 1306,61	0,06	42080,04 ± 1998,26 *	0,04	20479,9 ± 1838,54	0,08	63840 ± 3482,0 *	0,05	20280 ± 1581,7	0,07	19040 ± 1240,0	0,06	19347 ± 1327,8	0,06
IVN	22248 ± 1809,75	0,08	15034 ± 1079,84	0.07	22428,36 ± 1602,03	0.07	15230,42 ± 1175,93	0,07	28188 ± 2081,94 *	0,07	15278 ± 1047,33	0,06	72900 ± 2001,3 *	0,02	16070 ± 1135,8	0,07	15068 ± 1248,5	0,08	16437 ± 1167,9	0,07
LVN	20262 ± 1765,89	0,08	19474 ± 1442,89	0.07	21483 ± 1546,63	0.07	20829 ± 1514,62	0,07	36288 ± 2755,02 *	0,07	19475 ± 1158,74	0,05	67284 ± 3287,6 *	0,04	19030 ± 1412,8	0,07	19010 ± 1333,3	0,07	20753 ± 1187,8	0,05
SVN	32800 ± 2195 *	0,06	25543 ± 1487,89	0,05	33360 ± 2227,5 *	0,06	25743 ± 1498,75	0,05	45160 ± 3505 *	0,07	26457 ± 1131,17	0,04	77760 ± 4080,0 *	0,05	26423 ± 1512,9	0,05	25760 ± 1557,5	0,06	26677 ± 1087,7	0,04
**UVN**																				
MVN	50338,23 ± 3562,01 *	0,07	19560 ± 1147,01	0,05	53264,7 ± 3492,88 *	0,06	19080 ± 1332,14	0,06	103852,94 ± 1171,25 *	0,01	19400 ± 1097,73	0,05	109500 ± 1299,8 *	0,01	19580 ± 1289,6	0,06	19647,05 ± 1599,8	0,08	20232 ± 1210,4	0,05
IVN	30480 ± 2291,02 *	0,07	15607 ± 1337,36	0,08	34800 ± 3064,05 *	0,08	16314,07 ± 1456,57	0,08	70380 ± 4687,11 *	0,06	14791,5 ± 1129,48	0,07	76040 ± 3311,3 *	0,04	16212 ± 1187,9	0,07	15612 ± 863,4	0,05	15545 ± 997,7	0,06
LVN	36120 ± 3132,34 *	0,08	20174 ± 1762,73	0,08	37800 ± 2750,98 *	0,07	20565 ± 1287,56	0,06	42682 ± 1936,43 *	0,04	20166 ± 1264,21	0,06	83200 ± 3323,4 *	0,03	19534 ± 1627,8	0,08	21988 ± 1588,1	0,07	20635 ± 1172,0	0,05
SVN	78075 ± 5490 *	0,07	26983 ± 1879,67	0,06	98550 ± 6519,37*	0,06	25994 ± 1781,42	0,06	118875 ± 1214,37 *	0,01	26173 ± 1186,06	0,04	130275 ± 1397,5 *	0,01	26300 ± 1317,5	0,05	26150 ± 1156,7	0,04	26541 ± 1031,8	0,03

Table 6. Mean total GFAP immuno-positive cell numbers and CE of stereological analysis for estimation of total GFAP immuno-positive cells in the ipsilateral and contralateral vestibular nuclei complexes of the sham and the experimental groups of cats for each survival period tested. Values are mean ± SEM; CE: coefficient of error; GFAP: glial fibrillary acidic protein; D: day; IVN: inferior vestibular nucleus; LVN: lateral vestibular nucleus; MVN: medial vestibular nucleus; SVN: superior vestibular nucleus; TTX: tetrodoxin; UL: unilateral labyrinthectomy; UVN: unilateral vestibular neurectomy.
